# Intratumoral and peritumoral radiomics based on 2D ultrasound imaging in breast cancer was used to determine the optimal peritumoral range for predicting KI-67 expression

**DOI:** 10.1007/s40477-025-01049-0

**Published:** 2025-07-10

**Authors:** Wangxing Huang, Songming Zheng, Xiaoyan Zhang, Lina Qi, Min Li, Qinghua Zhang, Zhen Zhen, Xiuwei Yang, Changqin Kong, Dong Li, Guoyong Hua

**Affiliations:** 1 Graduate School of Qinghai University, Xining, China; 2 School of Computer Technology and Application, Qinghai University, Xining, China; 3 Interventional Ultrasound Department, Qinghai Provincial People’s Hospital, Xining, China

**Keywords:** Radiomics, Breast cancer, KI-67 prediction, Intratumoral and peritumoral, Machine learning

## Abstract

**Objectives:**

Currently, radiomics focuses on intratumoral regions and fixed peritumoral regions, and lacks an optimal peritumoral region taken to predict KI-67 expression. The aim of this study was to develop a machine learning model to analyze ultrasound radiomics features with different regions of peri-tumor fetch values to determine the optimal peri-tumor region for predicting KI-67 expression.

**Methods:**

A total of 453 breast cancer patients were included. They were randomly assigned to training and validation sets in a 7:3 ratio. In the training cohort, machine learning models were constructed for intra-tumor and different peri-tumor regions (2 mm, 4 mm, 6 mm, 8 mm, 10 mm), identifying the relevant Ki-67 features for each ROI and comparing the different models to determine the best model. These models were validated using a test cohort to find the most accurate peri-tumor region for Ki-67 prediction. The area under the receiver operating characteristic curve (AUC) was used to evaluate the performance of predicting KI-67 expression, and the Delong test was used to assess the difference between each AUC.SHAP (Shapley Additive Decomposition) was performed to analyze the optimal prediction model and quantify the contribution of major radiomics features.

**Results:**

In the validation cohort, the SVM model with the combination of intratumoral and peritumoral 6 mm regions showed the highest prediction effect, with an AUC of 0.9342.The intratumoral and peritumoral 6-mm SVM models showed statistically significant differences (*P* < 0.05) compared to the other models. SHAP analysis showed that peri-tumoral 6 mm features were more important than intratumoral features.

**Conclusion:**

SVM models using intratumoral and peritumoral 6 mm regions showed the best results in prediction of KI-67 expression.

## Introduce

Breast cancer is the most common malignant tumor in the world, and its Global Burden of Disease (GBD) has become a major threat to the health of the world's population, particularly women [1]. According to the Global Breast Cancer Statistical Report (GBCSR), there will be 2,261,000 new cases and 685,000 deaths worldwide in 2020, making breast cancer the number one malignant tumor in the world [2]. KI-67 is a nuclear protein involved in tumor cell proliferation [3]. KI-67 expression has also been used to classify luminal breast cancer into luminal A and luminal B types [4]. Studies have also shown that high KI-67 expression is an independent predictor of poorer recurrence-free survival in breast cancer patients [5]. With high expression of KI-67, breast cancer lesions have a more invasive and poorer prognosis, and patients with high expression of KI-67 should be treated more aggressively [6]. Currently, KI-67 is mainly obtained by hollow core needle biopsy(CNB),vacuum-assisted biopsy (VAB),or even more invasive excisional biopsy. These collection methods are invasive, costly, non-repeatable, and more prone to complications. In addition, conventional methods cannot dynamically and continuously detect changes in KI-67 after neoadjuvant chemotherapy [7]. Therefore, there is a current need for a noninvasive, reproducible,dynamic,and less costly method to evaluate KI-67 levels in breast cancer patients.

With the development of precision medicine, there is a growing interest in exploring potential biomarkers embedded in different types of medical images.Extracting high-throughput features from a large number of medical images and analyzing them with specific prognostic and diagnostic classifications is radiomics in a broad sense. Therefore,it is particularly important to rely on preoperative imaging images to build a predictive model for noninvasive prediction of KI-67 expression.Liu et al. used preoperative MRI images to predict KI-67 expression in breast cancer,with an area under the receiver operating characteristic curve (AUC) of 78.5% [8]. Zhu et al. in et al. used preoperative ultrasound images to predict KI-67 expression in breast cancer,with an area under the receiver operating characteristic curve (AUC) of 77% [9]. Previous studies have shown good results using either MRI/US images,but most studies had an AUC of 70–80%.It may be that all previous studies only considered intratumoral images of breast cancer and ignored the characteristics of peritumoral images.Previous studies have shown that the microenvironment surrounding breast cancer contains a variety of immune cells, blood vessels, and extracellular matrix, and that immune cell distribution and vascular survival may alter tumor progression and metastasis [10]. Jing et al. predicted an AUC of 88% for KI-67 expression using intratumoral and peritumoral ultrasound images of breast cancer [6]. However, the optimal size of the peritumoral region has not been systematically studied. Although some studies have investigated the range of values for the peritumoral area, they have been limited to MRI or X-ray imaging. There is no study based on 2D ultrasound images to determine the optimal range of peritumor area for predicting KI-67. Different value ranges of the peritumoral region extract different information about the tumor microenvironment. Determining the optimal range of peritumor values is essential for accurate prediction of KI-67.

Therefore, the purpose of this study is to establish a machine learning based on intratumoral regions and different peritumoral regions to explore the optimal peritumoral region size for KI-67 prediction which will improve the accuracy of preoperative assessments breast cancer.

## Materials and methods

### Patients selcetion

The study was approved by the hospital ethics committee (2024–064), and informed consent was waived for all enrolled patients. Patients who underwent preoperative ultrasound and subsequent surgical resection with pathological confirmation of breast cancer diagnosis from January 2015 to December 2023 were retrospectively analyzed. Exclusion criteria were (1) receipt of ablation or chemotherapy prior to ultrasound, (2) poor quality of ultrasound images, and (3) lack of a complete pathology report. Finally, 453patients were enrolled and randomized in a 7:3 ratio to training or validation cohorts. Complete pathologic, ultrasound, and clinical data were obtained from the case system. KI-67 expression was quantified according to the St. Gallen International Expert Consensus Guidelines. KI− 67 ≥ 14% was defined as high expression and < 14% as low expression.

### Image acquistion and segmentation

**Fig. 1:**
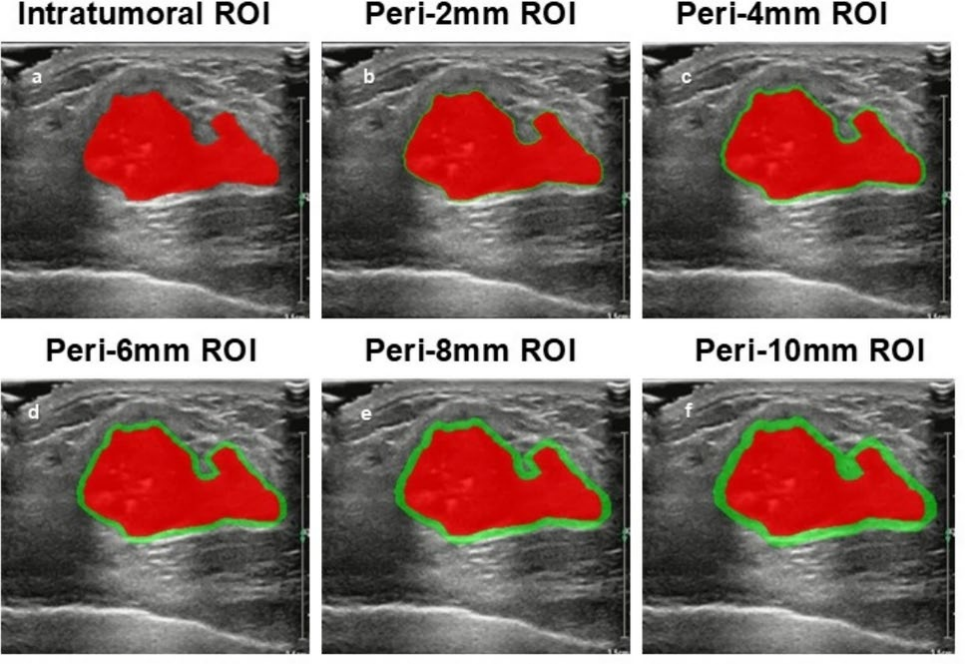
Intratumoral and peritumoral ROI acquisition is demonstrated.The red area represents the intratumoral area.The green area represents the extratumoral area.The labels *a*–*f* represent distinct regions of interest (ROIs): a denotes the intratumoral ROI of the breast cancer lesion, followed by *b, c, d, e*, and *f* representing concentric peri-tumoral regions generated by incremental expansions of 2 mm, 4 mm, 6 mm, 8 mm, and 10 mm outward from the tumor boundary, respectively

Breast ultrasound was performed according to a standard sweep protocol using a Philips EPIQ5 with an L12-3 high-frequency probe to acquire longitudinal and transverse ultrasound images, and the image with the largest diameter of the lesion was used as input and saved in NII format.Image resampling and grayscale normalization were performed to improve reliability prior to extracting radiomics features.The radiomics intra-tumor region of interest(ROI)were manually segmented by a physician with 10 years of experience in diagnostic ultrasound using the open-source imaging platform 3D Slicer software (v5.0.2).To capture the peritumor region, the intra-tumor region of interest (ROI-0) was expanded outward from the tumor boundaries by 2 mm, 4 mm, 6 mm, 8 mm,and 10 mm, respectively.The intra-tumor ROIs were then subtracted from the expanded ROI to obtain the peritumor ROI, resulting in a total of six different ROIs(0,0-2 mm,0-4 mm,0-6 mm, 0-8 mm, 0-10 mm). The partition of ROI is shown in Fig. 1.

### Radiomics feature extraction and selection

After accurately segmenting the intratumoral and various peritumoral regions, a standardized radiomics analysis platform was built using the Pyradiomics open-source toolkit in Python(https: //www.example.com en/latest/). A multi-scale analysis strategy was used to design the feature extractor and automatically extract radiomics features, 658 features were extracted for each ROI,and a total of 1316 features were extracted for the intra-tumor combined with different peri-tumor ranges of ROIs. The features of each ROI specifically include 120 raw image features:19 first-order features, which correspond to the symmetry,homogeneity, and local intensity distribution variations of the measured voxels.26 shape features (16 Shape2D features + 10 Shape3D features), which quantitatively characterize the 75 s- and higher-order texture features that reflect the spatial arrangement between gray levels of image voxels. The main categories include 24 Gray Level Cooccurrence Matrix (GLCM); 16 Gray Level Run Length Matrix(GLRLM); 16 Gray Level Size Zone Matrix(GLSZM); 5 Neighboring Gray Tone Difference Matrix(NGTDM); 14 Gray Level Dependence Matrix (GLDM).In addition,to increase the depth and complexity of the feature set, 538 s- and higher-order texture features are extracted by extending the feature expressiveness of the original image through various filtering methods. Local Binary Pattern (LBP) filtering:Construction of 8 × 8 neighborhood multi-scale LBP operator, extraction of 92-dimensional texture features.Wavelet transform: Adopt Daubechies-4 wavebase for multi-resolution analysis,generate 184-dimensional time–frequency domain feature logarithmic mapping: Enhancing the image contrast by log(1 + x) transformation,obtaining 276-dimensional intensity distribution features. Finally, the intra-tumor specific features and peri-tumor area association features were extracted, with a total of 133 features.Zone association features are extracted with a total of 1316 features, which significantly improves the discriminative ability of the features.

Then, a three-step approach was systematically used to select the best predictive features. First, the minimum redundant maximum relevance (mRMR) method was used to select the top 50 features most relevant to Ki-67 expression, then Spearman rank correlation was used to eliminate features with correlation coefficients greater than 0.85, and finally tenfold cross-validation combined with the least absolute shrinkage and selection operator (LASSO) method was used for non-zero feature selection. Spearman correlation was first used to eliminate features with correlation coefficients.

### Development and internal verification of ML models

In order to determine the optimal peritumor region for KI-67 prediction and to reduce the influence of different models on the results, a Support Vector Machine (SVM) prediction model based on multiregional feature fusion was constructed as an analytical tool in this study. The model is based on the combination of intra-tumor region (ROI-intra) and various peri-tumor regions (ROI-peri, distance 2–10 mm) for feature cascade composition.This multi-scale feature combination effectively overcomes the limitations of single region analysis. The data were first divided into training and validation sets by fivefold cross-validation in the model training phase, followed by the application of a Bayesian optimization algorithm to search for the optimal parameter combinations of the radial basis function (RBF) kernel function in the internal tuning phase, and finally the model stability was verified by repeating the process in three independent experiments. This process ensures strict assignment of data to the specified training and testing segments. The internal validation process uses receiver operating characteristic (ROC) curve analysis, area under the curve (AUC), and DeLong test to evaluate model performance.

### Interpretability of the optimal model

**Fig. 2:**
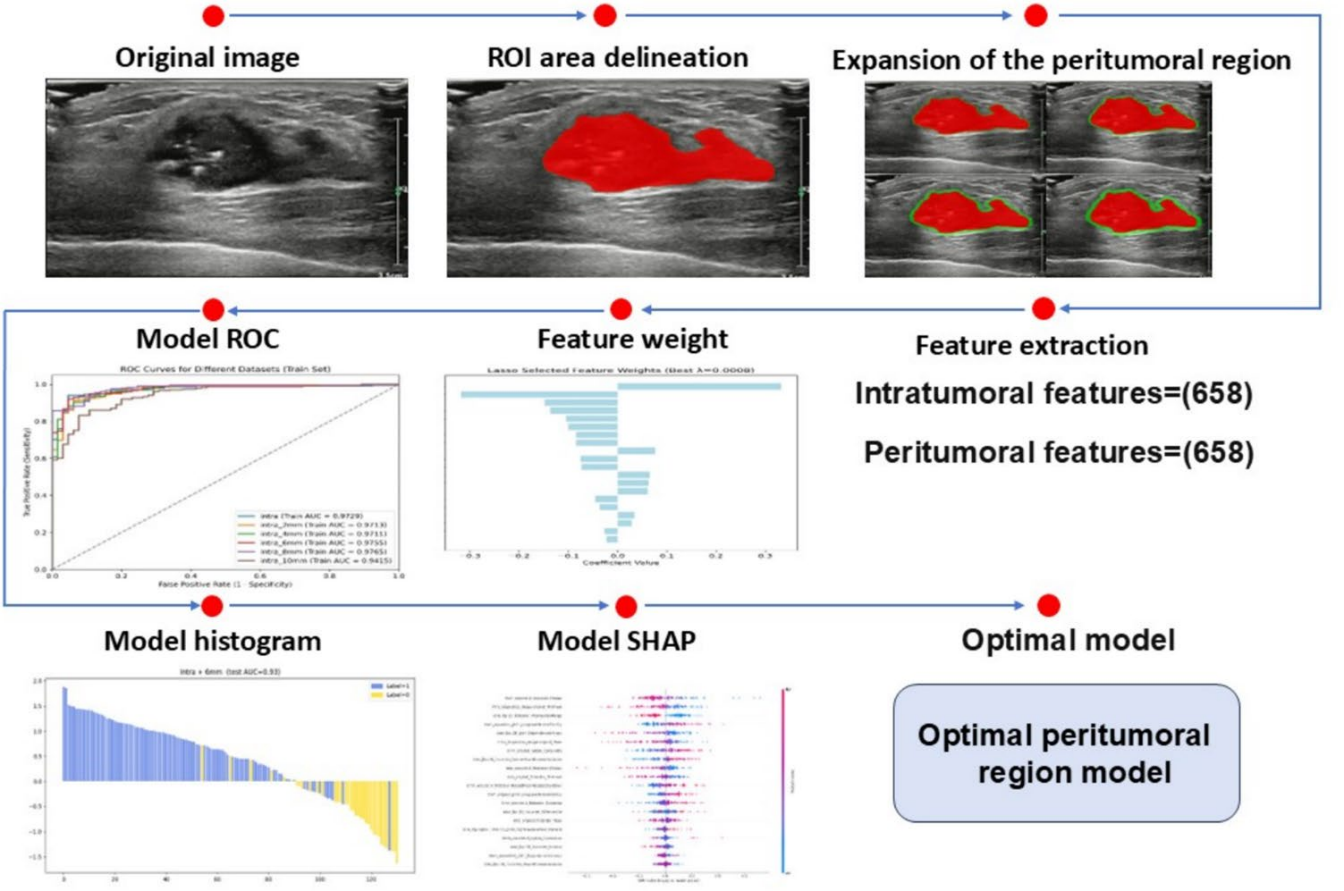
A detailed flow of the entire research process

Shapley Additive Explanations (SHAP) analysis was used to profile how well each feature predicted the model. The quantitative data provides a clear understanding of the impact of the extracted radiomics features on the prediction of KI-67 expression. The flowchart illustrates the process from image processing to model building, as shown in Fig. 2.

### Statistic analysis

All statistical analyses were performed with Python (version 3.8.20). Normality was tested using the Shapiro––Wilk method, with normally distributed data expressed as mean ± standard deviation and skewed data expressed as median interquartile range. Independent *t*-tests and chi-squared tests were used for comparisons between groups. The performance of predicting KI-67 expression was evaluated using the area under the curve (AUC). In addition, the DeLong method was used to compare the differences in AUC values between different combinations of regions.

## Result

### Patient characteristics

In this study, 453 female breast cancer patients with a mean age of 53.03 ± 3.53 years in the training cohort and 53.16 ± 3.44 years in the testing cohort were analyzed.386 patients (85.21%) showed high Ki-67 values in postoperative pathology. Table 1 shows that the training and test cohorts were similar in terms of demographic and clinical characteristics, with no significant differences observed (*p* > 0.05).

**Table 1:** Clinical characteristics comparison between training and validation cohorts

Characteristics	Training cohort	Validation cohort	*P*
Age(years)	53.03 ± 3.53	53.16 ± 3.44	0.724^a^
Menopausal status			0.317^b^
Premenopausal	129(40.57%)	48(35.56%)	
Postmenopausal	189(59.43%)	87(64.44%)	
Family history			0.980^b^
Absent	297(93.40%)	126(93.33%)	
Present	21(6.60%)	9(6.67%)	
Tumor diameter(CM)	2.70 ± 0.24	2.68 ± 0.23	0.575^a^
Location			0.866^b^
Left	170(53.46%)	71(52.59%)	
Right	148(46.54%)	64(47.41%)	
US-report LN status			0.380^b^
Negative	274(86.16%)	112(82.96%)	
Positive	44(13.84%)	23(17.04%)	
High KI-67	216(67.92%)	103(76.30%)	0.074^b^
Molecular subtypes			0.930^b^
Luminal A	96(30.19%)	42(31.11%)	
Luminal B	116(36.48%)	46(34.07%)	
HER-2 positive	76(23.90%)	32(23.71%)	
Triple negative	30(9.43%)	15(11.11%)	
BI-RADS			0.470^c^
4A	28(8.81%)	16(11.85%)	
4B	99(31.13%)	46(34.07%)	
4C	79(24.84%)	30(22.22%)	
5	112(35.22%)	43(31.85%)	

^a^for independent sample t-test, ^b^for chi-square test, and ^c^for Mann-Whitney U test

### Extraction and selection of radiomic features

**Fig. 3:**
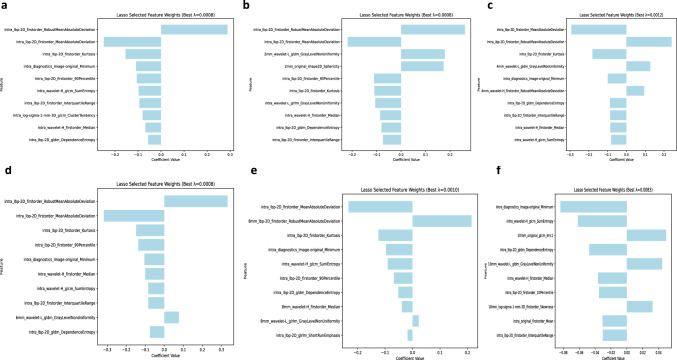
Radiomic features of KI-67 screened by Lasso for prediction. **a** Features of intratumoral model screening; **b** Features of intratumoral + peritumoral 2 mm model screening; **c** Features of intratumoral + peritumoral 4 mm model screening; **d** Features of intratumoral + peritumoral 6 mm model screening; **e** Features of intratumoral + peritumoral 8 mm model screening; **f** Features of intratumoral + peritumoral 10 mm model screening

In the training cohort,658,1316,1316,1316,1316,1316,radiomics features were identified by the process of segmenting the regions of interest of intratumoral,intratumoral + peritumoral 2 mm, intratumoral + peritumoral 4 mm, intratumoral + peritumoral 6 mm, intratumoral + peritumoral 8 mm, intratumoral + peritumoral 10 mm, intratumoral + peritumoral 10 mm, respectively, followed by stepwise application of mRMA, Sperman, and LASSO methods after screening the different regions of interest for 25, 27, 24, 24, 28, and 15 significant features related to KI-67 expression, respectively. The distribution of the features is shown in Fig. 3.

### ML model derivation and evaluation

**Fig. 4:**
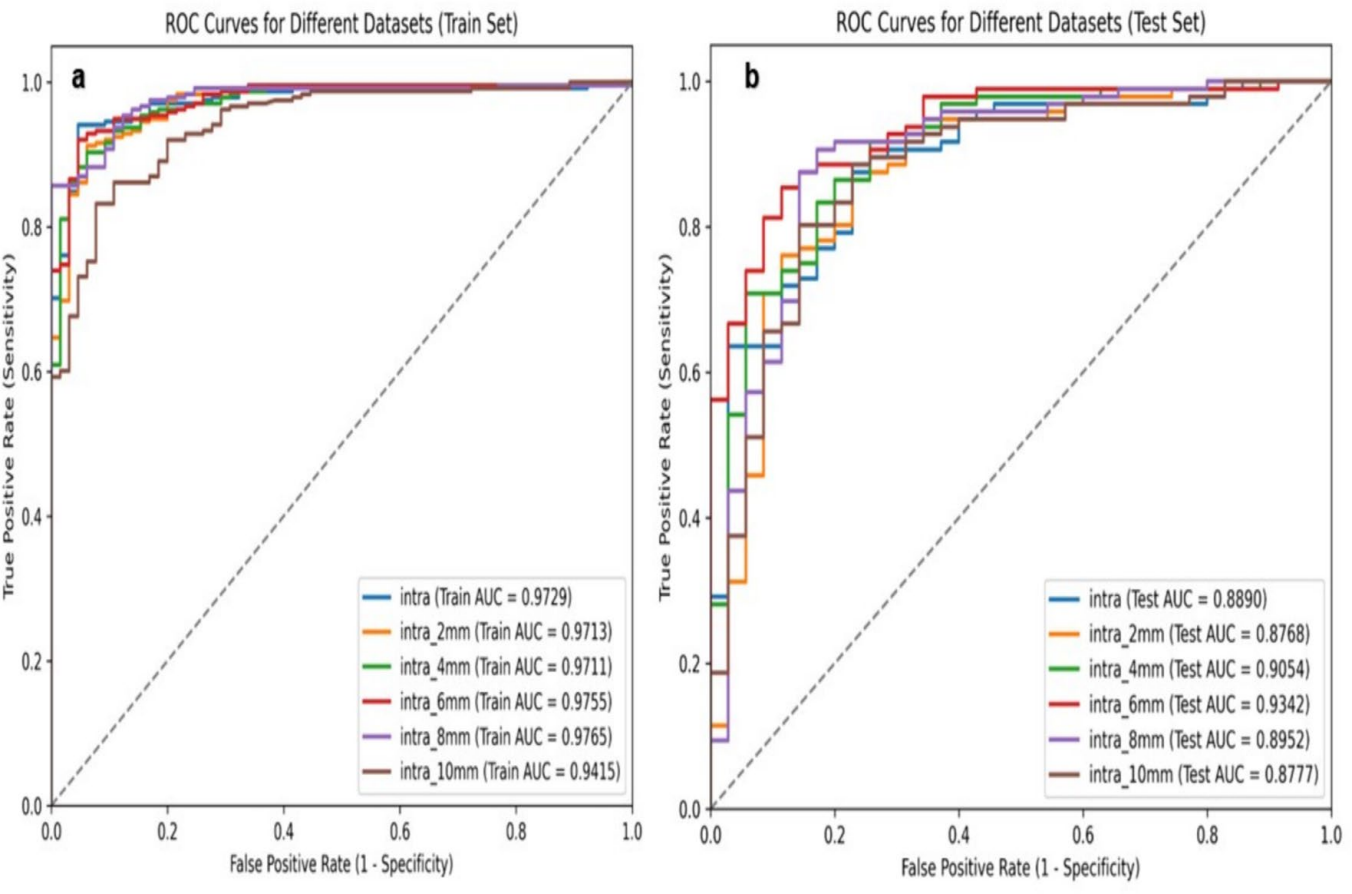
ROC curves of the model. **a** ROC curves of the training cohort; **b** ROC curves of the validation cohort

In order to select the best peritumor range for predicting KI-67 in breast cancer and to avoid differences between different machine learning models, we only applied SVM models. The AUC range for predicting KI-67 expression in the training cohort was 0.9415–0.9765. Intratumoral + peritumoral 6 mm and intratumoral + peritumoral 8 mm had the highest AUCs of 0.9755 and 0.9765, respectively. The predictive power of different peritumoral region models for KI-67 is shown in Table 2 and Fig. 4.

**Table 2:** :Predictive model performance for different ROIs in the Training Cohort

ROI	Model	AUC
Intra	SVM	0.9729
Intra + 2 mm	SVM	0.9713
Intra + 4 mm	SVM	0.9711
Intra + 6 mm	SVM	0.9755
Intra + 8 mm	SVM	0.9765
Intra + 10 mm	SVM	0.9415

### Model validation

An internal validation cohort was used to validate the predictive ability of the SVM models.The AUC for predicting KI-67 expression in the validation cohort ranged from 0.8768 to 0.9342. Intratumoral + peritumoral 6 mm achieved the highest AUC of 0.9342(95% CI 0.8342–0.9403)in the validation cohort of 6 models.Table 3 and Fig. 4 show the predictive power of the model in the validation queue.

**Table 3:** Predictive model performance for different ROIs in the Test Cohort

ROI	Model	AUC	AUC (95% CI)	Sensitivity	Specificity
Intra	SVM	0.8890	0.8890 (0.8044, 0.9031)	0.8958	0.7429
Intra + 2 mm	SVM	0.8768	0.8768 (0.8229, 0.8819)	0.8854	0.7143
Intra + 4 mm	SVM	0.9054	0.9054 (0.7972, 0.9190)	0.8646	0.8000
Intra + 6 mm	SVM	0.9342	0.9342 (0.8342, 0.9403)	0.8854	0.8286
Intra + 8 mm	SVM	0.8952	0.8952 (0.8289, 0.9085)	0.8958	0.8286
Intra + 10 mm	SVM	0.8777	0.8777 (0.7780, 0.8937)	0.8958	0.7429

### Comparison using delong test

**Fig. 5:**
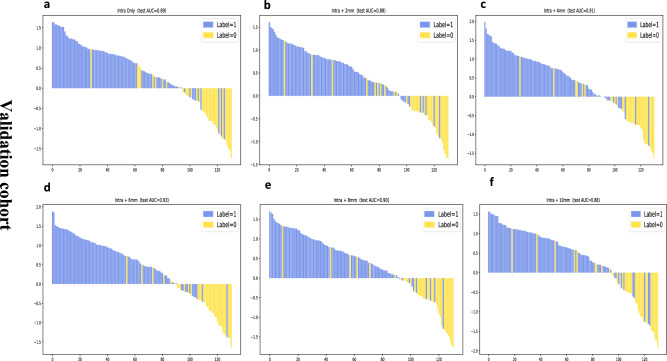
Use the Delong test in the validation cohort. **a** Delong test for intratumoral models; **b** Delong test for intratumoral + peritumoral 2 mm models; **c** Delong test for intratumoral + peritumoral 4 mm models; **d** Delong test for intratumoral + peritumoral 6 mm models; **e** Delong test for intratumoral + peritumoral 2 mm models; **f** Delong test for intratumoral + peritumoral 2 mm models

The Delong test is used to evaluate the differences between models. The results of the Delong test for the different models are shown in Fig. 5.The results showed statistically significant differences in the discriminative ability between the intratumoral and peritumoral 6-mm models and the other five models, as shown in Table 4.

**Table 4:** Delong test results of the 6 mm intratumoral + peritumoral model versus other models

Model	*P*
Intra + 6 mm *VS* Intra	0.027
Intra + 6 mm *VS* Intra + 2 mm	0.018
Intra + 6 mm *VS* Intra + 4 mm	0.017
Intra + 6 mm *VS* Intra + 8 mm	0.014
Intra + 6 mm *VS* Intra + 10 mm	0.021

### Interpretation of the optimal model

**Fig. 6:**
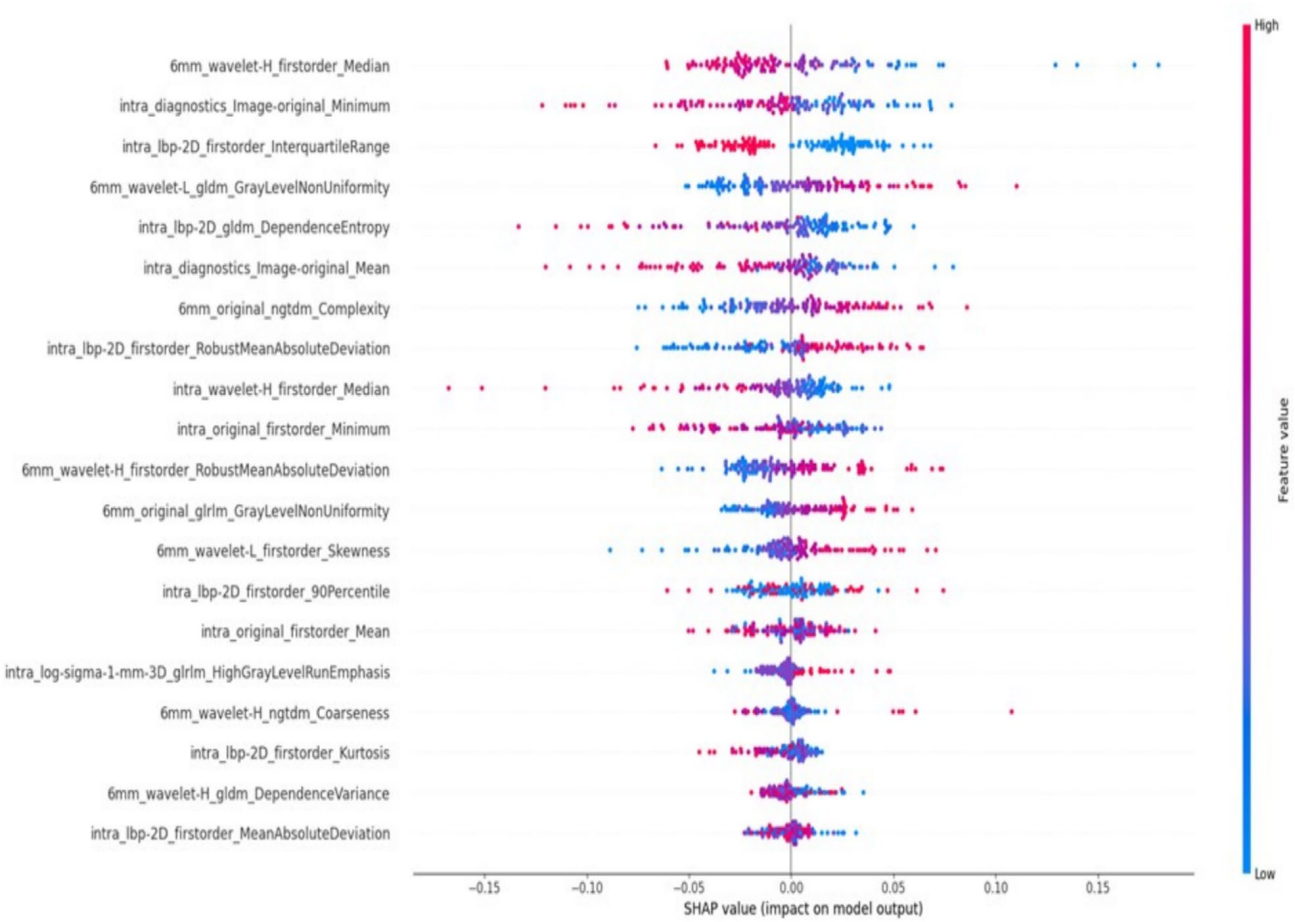
SHAP analysis of feature contributions for the SVM Model (ROI_0 + 6).The top 20 radiomics features are displayed, with colors indicating weights, red indicating high, and blue indicating low

The SVM model for ROI (0-6 mm) was interpreted using SHAP analysis to quantify the extent to which each feature contributed to the model. By calculating the absolute mean of the SHAP values, the cumulative influence of each variable was evaluated. The top 20 influences were listed according to the degree of contribution, and it was found that the most contributing influence was from peri-tumor 6 mm, and that two of the top four features were from peri-tumor 6 mm. This finding suggests that features from peri-tumor 6 mm exerted a more significant influence, emphasizing the importance of peri-tumor 6 mm in the prediction of KI-67 using 2D ultrasound images. The SHAP analysis is shown in Fig. 6.

## Discussion

Our study showed that the SVM model based on the combined intratumoral and peritumoral 6-mm region had the highest accuracy for the preoperative noninvasive prediction of KI-67 with an AUC of 0.9342, demonstrating the importance of the peritumoral 6-mm region in the preoperative noninvasive prediction of KI-67.The innovation of this study was the identification of the optimal range of 2D ultrasound images for predicting KI-67. Two-dimensional ultrasound imaging is widely used in clinical practice due to its simplicity and cost-effectiveness, and our findings will help clinicians to better assess breast cancer aggressiveness preoperatively, thereby improving patient outcomes.

US is convenient, cost-effective and highly reproducible compared to MRI, allowing clinicians to dynamically visualize the lesion site and evaluate the lesion from all angles. On the other hand, breast ultrasound does not produce ionizing radiation, making it a safe option for repeat imaging and follow-up, especially in individuals who may be more sensitive to radiation exposure. Recent studies have emphasized the importance of considering cumulative radiation dose in health care, making ultrasound a favorable option for diagnostic evaluation, especially in younger populations or those requiring frequent imaging [11].

Many studies in recent years have shown that radiomics can noninvasively predict KI-67 expression levels, but most of the studies were based on a single peritumoral image from X-ray or MRI, and the AUCs were between 0.7 and 0.8 [12, 13].The peritumoral region of breast cancer is closely associated with tumor aggressiveness,which is rich in lymphovascular infiltration, microvascular proliferation,and peritumoral stromal reaction [14]. Jing et al. reported for the first time internationally that an ultrasound imaging histology model based on intratumoral and peritumoral predicts KI-67 expression, and their results reached an AUC of 0.88 [6], which is higher than the previous single intratumoral model. However, there was no discussion on the optimal range of values for predicting KI-67 expression, and instead, the optimal peritumor range for predicting metastasis of anterior sentinel lymph nodes of breast cancer (3 mm) was taken.The different ranges of values contain different information and may affect the results of predicting KI-67 expression [15].When the feature extraction range was extended to 4 mm peri-tumor, the AUC increased to 0.905 relative to the intratumor image, suggesting that 4 mm peri-tumor contains features closely related to KI-67 expression. When the feature extraction range reached 6 mm, the AUC was optimal (0.934), suggesting that 6 mm of the peritumor contained more important features compared to 4 mm. Bin et al. showed that the optimal peritumor range for predicting KI-67 expression in breast cancer based on Automated Breast Volume Scanning images showed that the accuracy of KI-67 prediction was highest when the peritumor range was 6 mm [16], and our results showed that the KI-67 prediction accuracy was highest when the peritumor range was 6 mm. When the feature extraction range reaches 8 mm, the AUC drops to 0.895,the possible reason is that beyond a certain range,more interfering information is extracted,leading to a decrease in the performance of predicting KI-67 expression. After that, all models were subjected to Delong test and found to have good predictive ability.

The SHAP interpretation intuitively reflects the role of different features in the prediction model. From the results of the SHAP analysis, the features that contributed most to the model were the textural features of the peritumoral regions, suggesting that the expression of KI-67 is associated with the heterogeneity of the tumor periphery, which is consistent with the results of previous studies [17].In particular, these peritumoral regions usually exhibit complex cellular interactions and microenvironmental changes, which may reflect the invasiveness of tumors and thus become an important indicator for predicting Ki-67 levels as an important indicator of tumor aggressiveness [18, 19].

Although this study identified the optimal values of the peri-tumor area for two-dimensional ultrasound to predict KI-67 expression, it suffers from the following shortcomings. First, this was a single-center study, and although an internal validation cohort was established, external data will be needed to validate the performance of the model in the future. Second, this is a retrospective study, which may lead to some selection bias.

## Conclusion

In this study, we investigated the ability to predict KI-67 expression based on 2D ultrasound images of intratumoral regions combined with different peritumoral areas using an SVM model, and found that the intratumoral combined with the peritumoral 6-mm area achieved the highest prediction accuracy. The model showed consistent and reliable performance in both the training and validation sets, suggesting that it is expected to improve the accuracy of preoperative breast cancer assessment and provide more patient-friendly treatment options.

## Authors’contributions

Conception:Wangxing Huang,Songming Zheng,Dong Li,Guoyong Hua; study design:Wangxing Huang,Songming Zheng,Dong Li,Guoyong Hua;data collection:Wangxing Huang, Xiaoyan Zhang,Lina Qi,Min Li;Qinghua Zhang;Zhen Zhen;Xiuwei Yang;Changqin Kong; data analysis: Wangxing Huang,Songming Zheng; data interpretation: Wangxing Huang,Songming Zheng;manuscript writing: Wangxing Huang,Songming Zheng; manuscript editing: Wangxing Huang,Songming Zheng.All authors (s) read and approved the final manuscript.

## Data Availability

The datasets used and/or analyzed during the current study are available from the corresponding author upon reasonable request.

## Abbreviations

AUC: Area under the ROC curve
ROI: Region of interest
GLCM: Gray Level Cooccurrence Matrix
GLRLM: Gray Level Run Length Matrix
GLSZM: Gray Level Size Zone Matrix
NGTDM: Neighboring Gray Tone Difference Matrix
GLDM: Gray Level Dependence Matrix
LBP: Local Binary Pattern
mRMR: Minimum redundant maximum relevance
LASSO: Least absolute shrinkage and selection operator
SHAP: Shapley Additive Explanations
CNB: Core needle biopsy
VAB: Vacuum-assisted biopsy
RBF: Radial basis function
